# Association of a Medicare Advantage Posthospitalization Home Meal Delivery Benefit With Rehospitalization and Death

**DOI:** 10.1001/jamahealthforum.2023.1678

**Published:** 2023-06-25

**Authors:** Huong Q. Nguyen, Lewei Duan, Janet S. Lee, Thomas G. Winn, Annet Arakelian, Jaime Akiyama-Ciganek, Dan N. Huynh, Della D. Williams, Bing Han

**Affiliations:** 1 Department of Research and Evaluation, Kaiser Permanente Southern California, Southern California Permanente Medical Group, Pasadena; 2 Department of Health Systems Science, Kaiser Permanente Bernard J. Tyson School of Medicine, Pasadena, California; 3 Centers for Medicare and Medicaid Services, Washington, DC; 4 Kaiser Foundation Health Plan, Pasadena, California; 5 Department of Hospital Medicine, Southern California Permanente Medical Group, Pasadena; 6 Kaiser Permanente Bernard J. Tyson School of Medicine, Department of Clinical Science, Pasadena, California

## Abstract

**Importance:**

The 2018 Chronic Care Act allowed Medicare Advantage plans to have greater flexibility in offering supplemental benefits, such as meals and services, to address unmet needs of beneficiaries with certain chronic conditions. Based on earlier studies of community-based nutritional support, such programs may result in reduced use.

**Objective:**

To evaluate the association of a 4-week posthospitalization home-delivered meals benefit with 30-day all-cause rehospitalization and mortality in patients admitted for heart failure (HF) and other acute medical conditions (non-HF).

**Design, Setting, and Participants:**

In this cohort study, patients who received meals (the meals group) were compared with 2 controls: (1) no meals in the 2019 historical cohort who would have been eligible for the benefit (the no meals–2019 group) and (2) no meals in the 2021 and 2022 concurrent cohort who were referred but did not receive the meals due to unsuccessful contacts and active declines (the no meals–2021/2022 group). This study took place in a large integrated health care system in southern California among Medicare Advantage members with a hospitalization for HF or other acute medical conditions at 15 Kaiser Permanente hospitals discharged to home.

**Exposure:**

The exposure was receipt of at least 1 and up to 4 shipments of home-delivered meals (total of 56 to 84 meals) after hospital discharge.

**Main Outcomes and Measures:**

The main outcomes were 30-day all-cause composite rehospitalization and death.

**Results:**

A total of 4032 adults with admission to the hospital for HF (mean [SD] age, 79 [9] years; 1951 [48%] White; 2001 [50%] female) and 7944 with non-HF admissions (mean [SD] age, 78 [8] years; 3890 [49%] White; 4149 [52%] female) were included in the analyses. Unadjusted rates of 30-day death and rehospitalization for the meals, no meals–2019, and no meals–2021/2022 cohorts were as follows: HF: 23.3%, 30.1%, and 38.5%; non-HF: 16.5%, 22.4%, and 32.9%, respectively. For HF, exposure to meals was significantly associated with lower odds of 30-day death and rehospitalization compared with the no meals–2021/2022 cohort (OR, 0.55; 95% CI, 0.43-0.71; *P* < .001) but was not significant compared with the no meals–2019 cohort (OR, 0.86; 95% CI, 0.72-1.04; *P* = .12). For non-HF, exposure to meals was associated with significantly lower odds of 30-day death and rehospitalization when compared with the no meals–2019 (OR, 0.64; 95% CI, 0.52-0.79; *P* < .001) and the no meals–2021/2022 (OR, 0.48; 95% CI, 0.37-0.62; *P* < .001) cohorts.

**Conclusions and Relevance:**

In this cohort study, exposure to posthospitalization home-delivered meals was associated with lower 30-day rehospitalization and mortality; randomized clinical trials are needed to confirm these findings.

## Key Points

**Question:**

What is the association of a 4-week posthospitalization home-delivered meals benefit with the composite outcome of 30-day rehospitalization and death in members of Medicare Advantage?

**Findings:**

In this comparative cohort study including 4032 older adults with hospital admission for heart failure and 7944 with non–heart failure admission, the Medicare Advantage home-delivered meals benefit was associated with lower odds of 30-day rehospitalization and death.

**Meaning:**

Provision of nutritional support after hospitalization may improve outcomes in older adults.

## Teaser

This cohort study evaluates the association of a 4-week posthospitalization home-delivered meals benefit with 30-day all-cause rehospitalization and death in patients admitted for heart failure and other acute medical conditions.

## Introduction

The 2018 Chronic Care Act allowed Medicare Advantage (MA) plans to have greater flexibility in offering supplemental benefits such as meals, transportation, and in-home supports to address unmet needs of beneficiaries with certain chronic conditions. Nearly three-quarters of MA plans offered meals as a supplemental benefit in 2022,^1^ mostly driven by expectations of downstream cost savings based on findings from earlier observational studies of community-based nutrition programs^2,3^ and desires to maintain market parity in an increasingly competitive MA space.

Kaiser Permanente Southern California (KPSC) began offering home-delivered meals as a new MA supplemental benefit for eligible members on January 1, 2021. Patients hospitalized for heart failure (HF) were the primary target of the new base meals benefit (2 meals per day). This is because HF was considered a nutrition-sensitive condition, and, as such, patients could potentially benefit clinically from receiving convenient nutritional support during a vulnerable period after hospital discharge. A more generous buy-up meals benefit (3 meals per day) was also offered to a subset of the members of MA who were covered under selected employer group plans for any hospitalized condition. Thus, we sought to evaluate the outcomes of this new posthospitalization home-delivered meals benefit on 30-day all-cause rehospitalization and death in patients admitted for HF and all other acute medical conditions (non-HF).

## Methods

### Design

This was a cohort study conducted at 15 hospitals in KPSC with a cohort of MA members who received 4 weeks of home-delivered meals after hospital discharge to home from January 1, 2021, to January 31, 2022, and 2 no-meals comparators (2019 historical and 2021/2022 concurrent cohorts). We included 2 control comparators in an attempt to address the potential unobserved confounding biases inherent in either cohort. The 2019 historical cohort does not have the selection bias that is expected with the concurrent cohort of patients who did not receive the meals, for reasons that could be associated with either increased or decreased risk for the primary study outcome (eg, patients whom the vendor was unable to contact because they may have been too ill to answer the phone or patients who actively declined the meals because they or their family could prepare their meals). Alternatively, the historical cohort does not account for secular changes in acute care use because of the COVID-19 pandemic and other factors. Despite each comparator’s limitation, there is no obvious reason to believe that the 2 comparators share a common source of unobserved confounding bias. Given the limitations of both comparators, we expected the true association between exposure to the meals and the composite outcome of all-cause readmission and death to be somewhere in the middle, with the concurrent controls providing a more liberal estimate of the association and the historical controls providing a more conservative estimate. The present study design has the potential to overcome the limitations of using either 1 of the 2 comparators, as it allows for a more comprehensive comparison between the 2. The study was approved by the KPSC institutional review board (No. 10594). Informed consent was waived because the research was low risk, the research could not practically be conducted with individual consent, and the waiver did not adversely affect the rights and welfare of the participants. The study followed the Strengthening the Reporting of Observational Studies in Epidemiology (STROBE) reporting guideline.

### Sample

Patients were enrolled in the Kaiser Foundation MA plan either as individual members (70%) or through their employer groups, eg, state or county retirees (30%). All individual MA plan members hospitalized with a principal diagnosis of HF were eligible for the base, 2-meals-per-day benefit, whereas selected group MA plan members were eligible for the buy-up, 3-meals-per-day benefit, regardless of their hospital diagnosis. For this reason, the analytical cohorts were stratified by patients with a principal diagnosis of HF or all other acute medical conditions. Because the inpatient case managers who identified the patients with HF for the benefit had to rely on the current hospital diagnosis and were not trained to further validate the diagnosis, post hoc validation using the stringent criteria of at least 3 outpatient diagnoses or more than 1 inpatient principal discharge diagnosis, not including the index hospitalization, revealed that only 505 (68%) of the 742 patients met these criteria; nonetheless, we retained all patients in the HF cohort to reflect the clinical implementation of such new policies. Patients who met the benefits eligibility criteria as deemed by the inpatient case managers, were hospitalized at KPSC hospitals, were discharged home with home health or hospice, and whose meal referral occurred during the hospitalization were included in the analyses.

The 2 comparators included the following: (1) the no meals–2019 historical controls and (2) the no meals–2021/2022 concurrent controls who were referred but did not receive the meals due to unsuccessful contacts or active declines for various reasons. For HF, we did not include any further restrictions when identifying the no meals–2019 historical controls, whereas, for non-HF, we only included control members who were covered by the employer group plans that offered the buy-up benefit in 2021 and 2022.

### Data Collection

Depending on whether patients were enrolled in an individual or employer group plan, they received either 2 (base) or 3 (buy-up) meals per day for a maximum of 56 or 84 meals within 4 weeks of hospital discharge. Patients were eligible for 1 of the 2 benefits, not both, once a calendar year. For instance, if a patient was hospitalized for HF and was covered under an employer group plan that had purchased the buy-up benefit, the patient would receive 3 meals per day, whereas another patient hospitalized for HF and covered under an individual plan would receive 2 meals per day. Most of the patients with HF (86%; n = 638) received the base meal plan.

Patients selected meals from 7 menus offered by the vendor (Mom’s Meals, PurFoods LLC; eg, general wellness, heart-friendly, lower sodium, diabetes, and kidney) and received up to 4 meal shipments unless they canceled the meals. Patients were considered to be exposed to the meals if the vendor records showed that they received at least 1 meal shipment which could contain 14 (base) or 21 (buy-up) meals. Median (IQR) time from hospital discharge to receipt of the first shipment was 7 (5-9) days; a small number of patients received their meals after 30 days (n = 6 for HF and n = 7 for non-HF).

### Outcomes

The primary outcome was 30-day all-cause composite rehospitalization and death. Deaths and rehospitalizations were also analyzed separately. Sixty-day all-cause and HF-specific rehospitalizations were secondary outcomes. These data were extracted from the electronic medical record system and claims.

### Covariates

Sociodemographic characteristics (age, sex, race and ethnicity, neighborhood deprivation index), health care utilization in the prior year (acute and ambulatory care), clinical and behavioral characteristics (Elixhauser comorbidity index [measures comorbidity burden based on diagnostic codes],^4^ frailty index [patients were categorized into 3 frailty risk groups: low, medium, and high risk],^5^ ejection fraction, and exercise), and severity of the index admission (LACE [length of stay, age, comorbidities, prior emergency department visits] readmission score,^6^ laboratory acute physiology score [a severity of illness score calculated using data from prior 72 hours],^7^ length of stay, admission source, functional status at discharge, support after discharge and discharge disposition) were extracted from the electronic medical record. Medical centers were also included as fixed-effect covariates to account for potential clustering and unobserved site-level confounders.

### Statistical Analysis

We generated descriptive statistics to summarize the data. Missing covariates were generally assigned to the largest category; for example, patients without exercise data in the prior year were assigned as inactive, or in the case of small cell sizes for the race categories (ie, Asian, Native American/Alaska Native, Pacific Islander, multiracial, and missing), patients were combined for the multivariable analyses. We conducted intention-to-treat inverse probability of treatment weight logistic regression analyses to examine the association between exposure to home-delivered meals and the prespecified primary and secondary outcomes. For each comparator, we estimated the propensity scores by a multivariable logistic regression using the study group as a binary outcome and the same covariates as in the primary analysis. These models accounted for relevant covariates based on their associations with the exposure and outcome. Sensitivity analyses were performed using adjusted Fine and Gray proportional hazard models with death as a competing risk for all-cause and HF-specific readmission, using the same set of covariates as the main models.^8^ We used the competing risk model to check if estimates from the logistic regression models were sensitive to the competing risk of death vs rehospitalization; ie, death that occurred before a future potential rehospitalization would prevent such a rehospitalization event from happening. Because the competing risk model requires careful records of time-to-event for both the outcome and the exposure, and the meal delivery records do not have the level of rigor to fully support the time-to-event analysis, we used the logistic regression model as the main approach and the competing risk model as sensitivity check for competing outcome risks. Adjusted odds ratios (ORs) and hazard ratios (HRs), corresponding 95% CIs, and *P* values were reported. All *P*-value tests were 2-sided, and *P* < .05 was considered statistically significant. All analyses were conducted using SAS statistical software, version 9.4 for Windows (SAS Institute).

## Results

### Patient Characteristics

A total of 4032 adults with admission to the hospital for HF (mean [SD] age, 79 [9] years; 1951 [48%] White individuals; 2001 [50%] female individuals) and 7944 with non-HF admissions (mean [SD] age, 78 [8] years; 3890 [49%] White individuals; 4149 [52%] female individuals) were included in the analyses. The selection of the home-delivered meal cohort and the 2 comparators are summarized in the Figure. Baseline characteristics for the 3 cohorts stratified by whether patients had a principal diagnosis of HF vs all other acute medical conditions are summarized in Table 1 (see eTables 1 and 2 in Supplement 1 for the standardized mean differences across the covariates). For the non-HF cohort, the most common discharge diagnosis categories were infectious, circulatory, digestive, and respiratory diseases (eTable 3 in Supplement 1). Acute care use in the prior year was higher for patients with HF (72%-82%) vs non-HF (66%-75%) as was the Elixhauser comorbidity index (11.5-11.9 vs 9.4-10.2). The top 3 reasons for why eligible patients did not receive the home-delivered meals included no contact (37%; n = 362), decline with no reasons provided (17%; n = 169), and already have help with meal preparation or able to self-prepare meals (18%; n = 171) (see eTable 4 in Supplement 1).

**Figure.**
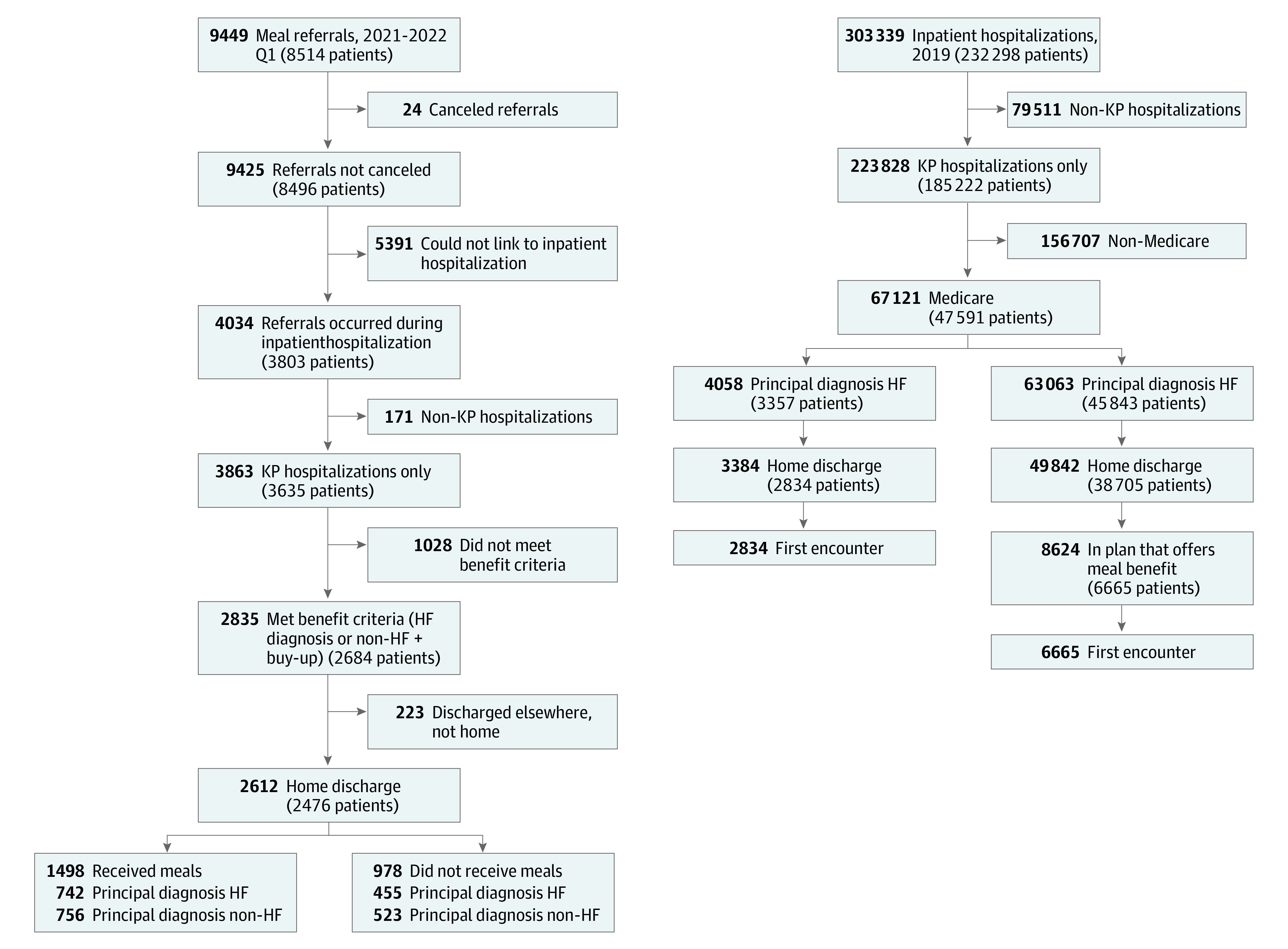
Sample Flow for Home-Delivered Meals and No Meals (2019 and 2021/2022) Cohorts The selection of the home-delivered meal cohort and the 2 comparators are summarized. Abbreviations: HF, heart failure; KP, Kaiser Permanente; Q1, quintile 1 for neighborhood deprivation (best quintile).

**Table 1.** Baseline Characteristics of Patients Receiving Home-Delivered Meals vs No Meals Comparators

Characteristic	No. (%)					
	HF meals (n = 742)	No meals comparators		Non-HF meals (n = 756)	No meals comparators	
		HF 2019 (n = 2834)	HF 2021/2022 (n = 455)		Non-HF 2019 (n = 6665)	Non-HF 2021/2022 (n = 523)
**Sociodemographic characteristics**						
Age, y, mean (SD)	78.8 (9.33)	79.0 (9.89)	79.8 (9.09)	77.9 (7.81)	77.3 (8.39)	78.9 (8.17)
Sex						
Female	379 (51)	1395 (49)	227 (50)	435 (58)	3435 (52)	279 (53)
Male	363 (49)	1439 (51)	228 (50)	321 (42)	3230 (48)	244 (47)
Race and ethnicity						
Asian	62 (8)	240 (8)	31 (7)	78 (10)	623 (9)	52 (10)
Black	144 (19)	394 (14)	70 (15)	202 (27)	1288 (19)	132 (25)
Native American/Alaska Native	1 (0)	6 (0)	0	2 (0)	19 (0)	1 (0)
Hispanic	233 (31)	745 (26)	120 (26)	169 (22)	1319 (20)	93 (18)
Pacific Islander	7 (1)	14 (0)	1 (0)	7 (1)	40 (1)	1 (0)
White	292 (39)	1427 (50)	232 (51)	290 (38)	3357 (50)	243 (46)
Multiracial	2 (0)	3 (0)	0	2 (0)	8 (0)	1 (0)
Missing	0	0	0	0	2 (0)	0
Marital status						
Partnered	350 (47)	1315 (46)	194 (43)	430 (57)	3744 (56)	288 (55)
Unpartnered	391 (53)	1517 (54)	261 (57)	326 (43)	2909 (44)	236 (45)
Missing	1 (0)	2 (0)	0	1 (0)	12 (0)	1 (0)
Spoken language: English	627 (85)	2439 (86)	400 (88)	722 (96)	6431 (96)	511 (98)
Insurance status						
Medicare	649 (87)	2504 (88)	403 (89)	753 (100)	6634 (100)	521 (100)
Dual (Medicare and Medi-Cal)	93 (13)	330 (12)	52 (11)	3 (0)	31 (0)	2 (0)
Neighborhood deprivation						
Q1 (best quintile)	110 (15)	619 (17)	88 (19)	155 (21)	1408 (21)	120 (23)
Q2	134 (18)	683 (19)	84 (18)	146 (19)	1373 (21)	107 (20)
Q3	140 (19)	680 (19)	80 (18)	150 (20)	1385 (21)	98 (19)
Q4	191 (26)	875 (24)	114 (25)	179 (24)	1489 (22)	123 (24)
Q5 (worst quintile)	167 (23)	715 (20)	88 (19)	126 (17)	991 (15)	74 (14)
Missing	0	4 (0)	1 (0)	0	0	1 (0)
Utilization in the year before the index hospitalization						
Any ED visits or inpatient/observation stays	532 (72)	2338 (82)	335 (74)	502 (66)	4497 (67)	390 (75)
Primary care (clinic, video, phone)	8.6 (7.99)	8.3 (7.97)	8.3 (7.61)	7.5 (5.63)	6.7 (7.25)	7.2 (5.92)
Specialty care (clinic, video, phone)	13.3 (14.55)	13.5 (14.07)	11.6 (12.53)	12.8 (14.12)	13.1 (13.73)	13.0 (14.51)
Clinical/behavioral						
Ejection fraction (EF)	49.6 (14.78)	47.3 (15.73)	49.2 (15.15)	57.0 (10.28)	56.7 (11.01)	56.9 (11.09)
EF <40	171 (23)	863 (30)	112 (25)	41 (5)	442 (7)	39 (7)
EF ≥40	543 (73)	1968 (69)	333 (73)	526 (70)	4705 (71)	371 (71)
Missing	28 (4)	3 (0)	10 (2)	189 (25)	1518 (23)	113 (22)
Elixhauser Comorbidity Index	11.5 (3.19)	11.9 (3.24)	11.9 (3.49)	9.9 (3.86)	9.4 (3.83)	10.2 (3.95)
Congestive HF	NA	NA	NA	315 (42)	2411 (36)	236 (45)
Dementia	82 (11)	390 (14)	66 (15)	99 (13)	1027 (15)	103 (20)
Other neurological disorders	215 (29)	796 (28)	144 (32)	244 (32)	1991 (30)	200 (38)
Chronic pulmonary disease	523 (70)	1993 (70)	297 (65)	472 (62)	3982 (60)	318 (61)
Diabetes	518 (70)	1927 (68)	306 (67)	442 (58)	3554 (53)	305 (58)
Kidney failure	573 (77)	2167 (76)	352 (77)	437 (58)	3689 (55)	299 (57)
Liver disease	163 (22)	600 (21)	107 (24)	187 (25)	1430 (21)	123 (24)
Cancer (any)	234 (32)	866 (31)	124 (27)	285 (38)	2561 (38)	214 (41)
Weight loss	248 (33)	1223 (43)	188 (41)	295 (39)	2475 (37)	229 (44)
Frailty index						
High	355 (48)	1630 (58)	239 (53)	356 (47)	3002 (45)	303 (58)
Medium	349 (47)	1103 (39)	201 (44)	328 (43)	3062 (46)	186 (36)
Low	38 (5)	101 (4)	15 (3)	72 (10)	601 (9)	34 (7)
Exercise in the prior year (median min/wk)						
Inactive (0 min/wk)	487 (66)	2014 (71)	268 (59)	424 (56)	3910 (59)	291 (56)
Insufficiently active (1-149 min/wk)	131 (18)	428 (15)	86 (19)	157 (21)	1303 (20)	105 (20)
Active (≥150 min/wk)	47 (6)	274 (10)	42 (9)	106 (14)	1179 (18)	72 (14)
Missing	77 (10)	118 (4)	59 (13)	69 (9)	273 (4)	55 (11)
**Characteristics of index admission**						
LACE readmission score						
<7	5 (1)	24 (1)	3 (1)	43 (6)	851 (13)	23 (4)
7-10	122 (16)	483 (17)	74 (16)	221 (29)	2008 (30)	137 (26)
≥11	615 (83)	2327 (82)	378 (83)	492 (65)	3806 (57)	363 (69)
Laboratory acute physiology score (LAPS2)	91.2 (27.82)	93.8 (30.61)	96.7 (30.18)	82.8 (44.81)	103.6 (29.96)	94.8 (32.11)
Length of stay	4.6 (3.02)	4.5 (3.80)	5.1 (3.81)	4.9 (4.08)	4.4 (4.85)	6.0 (7.43)
Code status						
Do not resuscitate/partial	188 (25)	861 (30)	157 (35)	114 (15)	1292 (19)	137 (26)
Full code	551 (74)	1966 (69)	297 (65)	637 (84)	5334 (80)	382 (73)
Missing	3 (0)	7 (0)	1 (0)	5 (1)	39 (1)	4 (1)
Functional status at discharge						
Nonambulatory	148 (20)	541 (19)	98 (22)	187 (25)	1507 (23)	171 (33)
Ambulatory	589 (79)	2263 (80)	355 (78)	563 (74)	5007 (75)	346 (66)
Missing	5 (1)	30 (1)	2 (0)	6 (1)	151 (2)	6 (1)
Admission source						
Home/clinic	656 (88)	2338 (82)	389 (85)	626 (83)	5369 (81)	431 (82)
Hospital/SNF and other	86 (12)	488 (17)	64 (14)	128 (17)	1267 (19)	91 (17)
Missing	0	8 (0)	2 (0)	2 (0)	29 (0)	1 (0)
Anticipated support after discharge						
Self	178 (24)	602 (21)	105 (23)	151 (20)	1103 (17)	90 (17)
Family/other	548 (74)	2056 (73)	335 (74)	584 (77)	4965 (74)	415 (79)
Missing	16 (2)	176 (6)	15 (3)	21 (3)	597 (9)	18 (3)
Discharge disposition						
Home/other	554 (75)	2182 (77)	351 (77)	474 (63)	3304 (50)	351 (77)
Home health/hospice	188 (25)	652 (23)	104 (23)	282 (37)	3361 (50)	104 (23)
Postdischarge outcomes						
30-d ED visits	119 (16)	532 (19)	94 (21)	131 (17)	1239 (19)	116 (22)
30-d Inpatient/observation stays	160 (22)	695 (25)	146 (32)	113 (15)	1194 (18)	130 (25)
30-d Alive, at home	28.1 (4.67)	26.8 (6.74)	25.0 (8.54)	28.6 (3.93)	27.5 (6.00)	25.4 (8.23)
All-cause outcomes						
Rehospitalization only	139 (19)	592 (21)	114 (25)	104 (14)	1069 (16)	102 (20)
Rehospitalization and death	21 (3)	103 (4)	32 (7)	9 (1)	125 (2)	28 (5)
Death only	13 (2)	157 (6)	29 (6)	12 (2)	299 (4)	42 (8)
None	569 (77)	1982 (70)	280 (62)	631 (83)	5172 (78)	351 (67)
HF-related hospitalizations						
HF rehospitalization only	63 (8)	255 (9)	59 (13)	NA	NA	NA
HF rehospitalization + death	6 (1)	31 (1)	12 (3)	NA	NA	NA
Death only	28 (4)	229 (8)	49 (11)	NA	NA	NA
None	645 (87)	2319 (82)	335 (74)	NA	NA	NA

Abbreviations: ED, emergency department; HF, heart failure as the principal diagnosis for the index admission; LACE readmission score, length of stay, age, comorbidities, prior emergency department visits; NA, not applicable; non-HF, all other acute medical conditions; SNF, skilled nursing facility.

### Primary Outcome

Unadjusted rates of 30-day death and rehospitalization for the meals, no meals–2019, and no meals–2021/2022 cohorts were as follows: HF: 23.3% (n = 173), 30.1% (n = 852), and 38.5% (n = 175); non-HF: 16.5% (n = 125), 22.4% (n = 1493), and 32.9% (n = 172), respectively (see eFigure 1 in Supplement 1).

For HF, exposure to meals was significantly associated with lower odds of 30-day death and rehospitalization compared with the no meals–2021/2022 cohort (OR, 0.55; 95% CI, 0.43-0.71; *P* < .001) but was not statistically significant compared with the no meals–2019 cohort (OR, 0.86; 95% CI, 0.72-1.04; *P* = .12) (Table 2).

**Table 2.** Readmission and Mortality for Home-Delivered Meals vs No Meals Comparators

Variable	All-cause death		All-cause readmission and death	
	OR (95% CI)	*P* value	OR or HR (95% CI)	*P *value
**Principal discharge diagnosis: heart failure (HF)**				
**30-d Outcomes**				
HF meals vs HF no meals–2019^a^	0.53 (0.37-0.77)	<.001	OR, 0.86 (0.72-1.04)	.12
HF meals vs HF no meals–2021/2022^a^	0.37 (0.23-0.60)	<.001	OR, 0.55 (0.43-0.71)	<.001
**Time to the first readmission**				
HF meals vs HF no meals–2019^b^	All-cause readmission		HR, 0.93 (0.80-1.08)	.35
HF meals vs HF mo meals–2021/2022^b^			HR, 0.70 (0.57-0.86)	<.001
HF meals vs HF no meals–2019^b^	HF-only readmission		HR, 1.08 (0.85-1.37)	.51
HF meals vs HF no meals–2021/2022^b^			HR, 0.67 (0.49-0.91)	.01
**Principal discharge diagnosis: all other medical conditions**				
**30-d Outcomes**	**All-cause death**		**All-cause readmission and death**	
Non-HF meals vs no meals–2019^c^	0.38 (0.24-0.61)	<.001	OR, 0.64 (0.52-0.79)	<.001
Non-HF meals vs no meals–2021/2022^c^	0.26 (0.16-0.42)	<.001	OR, 0.48 (0.37-0.62)	<.001
**Time to the first readmission**				
Non-HF meals vs no meals–2019^d^	All-cause readmission		HR, 0.86 (0.73-1.00)	.05
Non-HF meals vs no meals–2021/2022^d^			HR, 0.72 (0.57-0.89)	.003

Abbreviations: HR, hazard ratio; OR, odds ratio.

^a^ Inverse probability of treatment weighting logistic regression models adjusted for age, sex, race and ethnicity, neighborhood deprivation index, prior year acute care utilization, Elixhauser co-morbidity index, ejection fraction, dementia, frailty index, characteristics of the index hospitalization (LACE readmission risk score, length of stay, laboratory acute physiology score, code status, admission source, functional status at discharge, support after discharge, and discharge disposition), and hospital site.

^b^ Fine and Gray proportional hazards model with death as a competing risk adjusted for the same set of variables as listed previously.

^c^ Inverse probability of treatment weighting logistic regression models adjusted for age, sex, race and ethnicity, neighborhood deprivation index, prior year acute care and ambulatory utilization, dementia, frailty index, exercise in the prior year, characteristics of the index hospitalization (LACE readmission risk score, length of stay, laboratory acute physiology score, code status, admission source, functional status at discharge, support after discharge, and discharge disposition), and hospital site.

^d^ Fine and Gray proportional hazards model with death as a competing risk adjusted for the same set of variables listed previously.

For non-HF, exposure to meals was associated with significantly lower odds of 30-day death and rehospitalization when compared with the no meals–2019 (OR, 0.64; 95% CI, 0.52-0.79; *P* < .001) and no meals–2021/2022 (OR, 0.48; 95% CI, 0.37-0.62; *P* < .001) cohorts.

### Secondary Outcomes

For patients with and without HF, there were no significant associations between meals exposure and time to the first all-cause or HF-specific rehospitalization compared with the no meals–2019 cohort (Table 2). However, there were significant associations between meals exposure and time to the first all-cause readmission (with HF: HR, 0.70; 95% CI, 0.57-0.86; *P* < .001; without HF: HR, 0.72; 95% CI, 0.57-0.89; *P* = .003) and HF-only readmission (HR, 0.67; 95% CI, 0.49-0.91; *P* = .01) compared with the no meals–2021/2022 cohort.

The positive association of the meals exposure with the composite outcome persisted, albeit slightly attenuated, into the 60-day postdischarge period for the patients without HF (no meals–2019 cohort: OR, 0.74; 95% CI, 0.62-0.88; *P* < .001; no meals–2021/2022 cohort: OR, 0.56; 95% CI, 0.44-0.72; *P* < .001) (see eTables 5 and 6 in Supplement 1). For patients with HF, the 60-day results were similar to that of the 30-day results, wherein the meals exposure showed a stronger association with the composite outcome compared with the no meals–2021/2022 cohort (OR, 0.61; 95% CI, 0.48-0.77; *P* < .001) vs the no meals–2019 cohort (OR, 0.89; 95% CI, 0.75-1.05; *P* = .16).

## Discussion

In this cohort study, we found that exposure to an MA supplemental benefit that offers up to 4 weeks of posthospitalization home-delivered meals was associated with lower odds of all-cause rehospitalization and death 30 days after hospital discharge for HF and other medical conditions compared with both a historical and concurrent control cohort. The positive outcome was mostly associated with lower mortality vs rehospitalization. While the survival benefits of nutritional support were unexpected based on prior observational studies,^2,3^ the present findings are aligned with a recent randomized clinical trial of 10 weeks of medically tailored meals (MTM) for adults with nutrition-sensitive conditions, eg, HF, diabetes, or chronic kidney disease, wherein MTM was significantly associated with 36% lower all-cause mortality at 90 days after discharge but did not reduce risk of all-cause rehospitalization compared with usual care.^9^ This trial of nearly 2000 patients who were on average 10 years younger than the current study (mean age of 68 years vs 78 years) was conducted in another region of Kaiser Permanente before the health plan implemented the benefit in 2021. The consistent survival benefit associated with the meals exposure in both the HF and non-HF cohort with the 2 no-meals comparators suggests that the present findings are not spurious and warrants additional confirmation.

A key difference between the present study and that of Go and colleagues^9^ from the matched comparisons performed by Berkowitz et al^2,3^ of Medicare-Medicaid beneficiaries was that meals were provided at outpatient referrals based on individuals’ nutritional risks vs in response to a triggering event like a hospitalization. These seminal papers informed a recent simulation study that projected substantial reductions in use and costs with national implementation of MTM for patients with diet-sensitive conditions and activity limitations.^10^ Reconciling these divergent findings will be important because MA plans may need to consider whether a triggering acute event is required to initiate meals to support recovery or whether more proactive, population-based screening and preemptive nutrition support achieve the greatest effect.

Another important unanswered question in the field is the number of meals and duration of nutritional support that is needed to achieve outcomes of importance to patients, health systems, and payers. While this home-delivered meals benefit was intended to be a short-term bridge for patients during a vulnerable period after discharge from the hospital, it was encouraging to see this association persisting into the 60-day period, especially for the non-HF cohort. It is unclear if the more robust outcomes observed in the non-HF vs the HF cohort were due to the larger number of meals (84 vs 56), a reflection of the clinical conditions that are more amenable to nutritional support, or some other factors. Prior nutritional support studies have provided anywhere from 10 to 21 meals per week for as short as 4 weeks and up to 8 months.^2,3,9,10,11,12^

### Limitations

Although there are several strengths to this study including a highly diverse cohort, a few limitations are worth noting. Due to the nature of the supplemental benefit, all eligible patients were offered meals, and thus, we had to rely on an observational comparative design with its inherent selection bias, residual confounding, and temporal threats to validity. As expected, the outcome of the meals exposure was consistently stronger when compared to the concurrent control cohort vs the historical cohort, which, as we have acknowledged earlier, is likely due to selection bias and residual confounding. Another notable limitation was the misclassification of HF and the heterogeneity of the non-HF cohort and consequently, small sample sizes that did not allow for subgroup analyses of clinical populations that might be more or less responsive to nutritional support. Ascertainment of deaths may be incomplete due to our reliance on membership and clinical records, but underascertainment would apply equally to both the meals and concurrent controls. We conducted intention-to-treat analyses because we could not feasibly account for whether patients ate the meals. Our analysis of the HF cohort included a combination of patients who received 2 meals per day (86%; n = 638) and 3 meals per day (14%; n = 104), which could have introduced heterogeneity in the exposure. Implementation of the meal referral was unreliable and often occurred after the hospitalization making it challenging to anchor the index date and thus, the analytical sample was far smaller than the number of patients who received the meals.

## Conclusions

In this study of an ethnically diverse cohort of MA members, exposure to home-delivered meals in the 4 weeks after a hospitalization was significantly associated with lower odds of 30-day rehospitalization and mortality; the positive outcome was mostly associated with reductions in mortality vs rehospitalization. Additional prospective clinical studies are needed to confirm these findings and to better understand the mechanisms for improved survival with posthospitalization nutritional support.

## Notes

Supplement 1. **eTable 1.** Heart Failure Cohort, Before and After Inverse Probability of Treatment Weight **eTable 2.** Non-Heart Failure Cohort, Before and After Inverse Probability of Treatment Weight **eTable 3.** Major Diagnostic Categories for the Non-Heart Failure Cohort **eTable 4.** Reasons for Why Eligible Patients Did Not Receive Home-Delivered Meals **eTable 5.** Secondary Descriptive Outcomes at 60-days Post-Discharge **eTable 6.** Secondary Outcomes at 60-days Post-Discharge **eFigure 1.** Days to Rehospitalization and Death

Supplement 2. Data Sharing Statement
