# Supplementary material for: Association of a Medicare Advantage Posthospitalization Home Meal Delivery Benefit With Rehospitalization and Death
Source: JAMA Health Forum. 2023 Jun 25;4(6):e231678. doi: 10.1001/jamahealthforum.2023.1678 (PMC10291360; doi:10.1001/jamahealthforum.2023.1678)
Supplement: Supplement 1. — eTable 1. Heart Failure Cohort, Before and After Inverse Probability of Treatment Weight eTable 2. Non-Heart Failure Cohort, Before and After Inverse Probability of Treatment Weight eTable 3. Major Diagnostic Categories for the Non-Heart Failure Cohort eTable 4. Reasons for Why Eligible Patients Did Not Receive Home-Delivered Meals eTable 5. Secondary Descriptive Outcomes at 60-days Post-Discharge eTable 6. Secondary Outcomes at 60-days Post-Discharge eFigure 1. Days to Rehospitalization and Death [file jamahealthforum-e231678-s001.pdf]

Supplemental Online Content

Nguyen HQ, Duan L, Lee JS, et al. Association of a Medicare Advantage posthospitalization home meal delivery benefit with rehospitalization and death. *JAMA Health Forum.* 2023;4(6):e231678. doi:10.1001/jamahealthforum.2023.1678

eTable 1. Heart Failure Cohort, Before and After Inverse Probability of Treatment Weight

eTable 2. Non-Heart Failure Cohort, Before and After Inverse Probability of Treatment Weight

eTable 3. Major Diagnostic Categories for the Non-Heart Failure Cohort

eTable 4. Reasons for Why Eligible Patients Did Not Receive Home-Delivered Meals

eTable 5. Secondary Descriptive Outcomes at 60-days Post-Discharge

eTable 6. Secondary Outcomes at 60-days Post-Discharge

eFigure 1. Days to Rehospitalization and Death

This supplemental material has been provided by the authors to give readers additional information about their work.

© 2023 Nguyen HQ et al. *JAMA Health Forum.*

eTable 1. Heart Failure Cohort, Before and After Inverse Probability of Treatment Weight

		No Meals Comparators		Meals vs. No Meals-2019		Meals vs. No Meals-2021/2022	
	**HF Meals n=742**	**HF 2019 n=2834**	**HF 2021/2022 n=455**	**Un-adjusted Std Diff**	**Adjusted Std Diff**	**Un-adjusted Std Diff**	**Adjusted Std Diff**
**Socio-Demographics**							
Age	78.8 (9.33)	79.0 (9.89)	79.8 (9.09)	-0.02	0.01	-0.11	0.00
Female	379 (51%)	1395 (49%)	227 (50%)	0.04	0.01	0.02	0.00
Race/Ethnicity							
Asian/Other/Multi/Unknown	73 (10%)	268 (9%)	33 (7%)	0.01	-0.02	0.09	-0.01
Black	144 (19%)	394 (14%)	70 (15%)	0.15	0.02	0.11	0.01
Hispanic	233 (31%)	745 (26%)	120 (26%)	0.11	-0.01	0.11	0.01
White	292 (39%)	1427 (50%)	232 (51%)	-0.22	0.01	-0.24	-0.01
Neighborhood deprivation (worst quintile)	167 (23%)	548 (19%)	88 (19%)	0.08	-0.02	0.08	0.01
**Utilization in year prior to index**							
Any ED visits or inpatient/observation stays	532 (72%)	2338 (82%)	335 (74%)	-0.26	0.00	-0.04	-0.02
**Clinical/Behavioral**							
Ejection fraction (EF)	49.6 (14.78)	47.3 (15.73)	49.2 (15.15)				
EF <40	171 (23%)	863 (30%)	112 (25%)	0.15	0.10	0.03	0.04
EF >=40	543 (73%)	1968 (69%)	333 (73%)	-0.15	0.00	-0.03	0.00
Missing	28 (4%)	3 (0%)	10 (2%)				
Elixhauser Comorbidity Index	11.5 (3.19)	11.9 (3.24)	11.9 (3.49)	-0.11	0.01	-0.11	0.01
Dementia	82 (11%)	390 (14%)	66 (15%)	-0.08	-0.02	-0.10	0.00
Frailty index							
High	355 (48%)	1630 (58%)	239 (53%)	-0.19	0.01	-0.09	0.00
Medium	349 (47%)	1103 (39%)	201 (44%)	0.16	-0.02	0.06	0.00
Low	38 (5%)	101 (4%)	15 (3%)	0.08	0.01	0.08	0.01
**Characteristics of index admission**							
LACE readmission score							
<7	5 (1%)	24 (1%)	3 (1%)	-0.02	-0.01	0.00	-0.01

© 2023 Nguyen HQ et al. *JAMA Health Forum.*

		No Meals Comparators		Meals vs. No Meals-2019		Meals vs. No Meals-2021/2022	
	**HF Meals n=742**	**HF 2019 n=2834**	**HF 2021/2022 n=455**	**Un-adjusted Std Diff**	**Adjusted Std Diff**	**Un-adjusted Std Diff**	**Adjusted Std Diff**
7-10	122 (16%)	483 (17%)	74 (16%)	-0.02	-0.03	0.00	0.01
11+	615 (83%)	2327 (82%)	378 (83%)	0.02	0.03	-0.01	0.00
Laboratory acute physiology score (LAPS2)	91.2 (27.82)	93.8 (30.61)	96.7 (30.18)	-0.06	-0.01	-0.14	0.01
Length of stay	4.6 (3.02)	4.5 (3.80)	5.1 (3.81)	0.03	0.03	-0.14	0.00
Code status							
DNR/partial	188 (25%)	861 (30%)	157 (35%)	-0.11	0.04	-0.20	0.00
Full Code/Missing	554 (75%)	1973 (70%)	298 (65%)	0.11	-0.04	0.20	0.00
Functional status at discharge							
Non-ambulatory	148 (20%)	541 (19%)	98 (22%)	0.02	-0.04	-0.04	0.01
Ambulatory	594 (80%)	2293 (81%)	357 (78%)	-0.02	0.04	0.04	-0.01
Admission source							
Home/Clinic	656 (88%)	2338 (82%)	389 (85%)	0.17	-0.01	0.09	0.00
Hospital/SNF & Other	86 (12%)	496 (18%)	66 (15%)	-0.17	0.01	-0.09	0.00
Anticipated support post discharge							
Self	178 (24%)	602 (21%)	105 (23%)	0.07	0.00	0.02	0.00
Family/Other/Missing	564 (76%)	2232 (79%)	350 (77%)	-0.07	0.00	-0.02	0.00
Discharge disposition							
Home/other/missing	554 (75%)	2182 (77%)	351 (77%)	0.05	-0.03	0.06	-0.01
Home health/hospice	188 (25%)	652 (23%)	104 (23%)	-0.05	0.03	-0.06	0.01

© 2023 Nguyen HQ et al. *JAMA Health Forum.*

eTable 2. Non-Heart Failure Cohort, Before and After Inverse Probability of Treatment Weight

		No Meals Comparators		Non-HF Meals vs. No Meals-2019		Non-HF Meals vs. No Meals-2021/2022	
	Non-HF Meals n=756	Non-HF 2019 n=6665	Non-HF 2021/2022 n=523	Un-adjusted Std Diff	Adjusted Std Diff	Un-adjusted Std Diff	Adjusted Std Diff
**Socio-Demographics**							
Age	77.9 (7.81)	77.3 (8.39)	78.9 (8.17)	0.07	0.00	-0.12	-0.01
Female	435 (58%)	3435 (52%)	279 (53%)	0.12	0.04	0.08	0.01
Race/Ethnicity							
Asian/Other/Multi/Unknown	95 (13%)	661 (10%)	55 (10%)	0.07	-0.01	0.06	0.00
Black	202 (27%)	1288 (19%)	132 (25%)	0.18	0.01	0.03	0.00
Hispanic	169 (22%)	1319 (20%)	93 (18%)	0.06	-0.01	0.11	0.01
White	290 (38%)	3357 (50%)	243 (46%)	-0.24	0.01	-0.16	-0.02
Neighborhood deprivation (worst quintile)	126 (17%)	991 (15%)	74 (14%)	0.05	0.04	0.07	0.02
**Utilization in year prior to index**							
Any ED visits or inpatient/observation stays	502 (66%)	4497 (67%)	390 (75%)	-0.02	0.04	-0.18	-0.01
Primary care (clinic, video, phone)	7.5 (5.63)	6.7 (7.25)	7.2 (5.92)	0.14	0.07	0.05	-0.01
Specialty care (clinic, video, phone)	12.8 (14.12)	13.1 (13.73)	13.0 (14.51)	-0.02	-0.01	-0.01	0.02
**Clinical/Behavioral**							
Ejection fraction (EF)	57.0 (10.28)	56.7 (11.01)	56.9 (11.09)	-	-	-	-
EF <40	41 (5%)	442 (7%)	39 (7%)	-	-	-	-
EF >=40	526 (70%)	4705 (71%)	371 (71%)	-	-	-	-
Missing	189 (25%)	1518 (23%)	113 (22%)	-	-	-	-
Frailty index							
High	356 (47%)	3002 (45%)	303 (58%)	0.05	0.03	-0.22	0.00
Medium	328 (43%)	3062 (46%)	186 (36%)	-0.06	-0.05	0.15	-0.02
Low	72 (10%)	601 (9%)	34 (7%)	0.03	0.04	0.12	0.03
Exercise in prior year (median mins/wk)							
Inactive(0mins/wk) & missing	564 (76%)	2132 (75%)	346 (66%)	0.05	-0.02	-0.02	0.01

© 2023 Nguyen HQ et al. *JAMA Health Forum.*

		No Meals Comparators		Non-HF Meals vs. No Meals-2019		Non-HF Meals vs. No Meals-2021/2022	
	**Non-HF Meals n=756**	**Non-HF 2019 n=6665**	**Non-HF 2021/2022 n=523**	**Un-adjusted Std Diff**	**Adjusted Std Diff**	**Un-adjusted Std Diff**	**Adjusted Std Diff**
Insufficiently Active(1-149mins/wk)	131 (18%)	428 (15%)	105 (20%)	0.03	-0.02	0.02	0.01
Active(>=150mins/wk)	47 (6%)	274 (10%)	72 (14%)	-0.10	0.05	0.01	-0.02
**Characteristics of index admission**							
LACE readmission score							
<7	43 (6%)	851 (13%)	23 (4%)	-0.25	-0.08	0.06	-0.01
7-10	221 (29%)	2008 (30%)	137 (26%)	-0.02	0.02	0.07	0.01
11+	492 (65%)	3806 (57%)	363 (69%)	0.16	0.02	-0.09	-0.01
Laboratory acute physiology score (LAPS2)	82.8 (44.81)	103.6 (29.96)	94.8 (32.11)	0.06	0.04	-0.19	0.00
Length of stay	4.9 (4.08)	4.4 (4.85)	6.0 (7.43)	0.11	-0.03	-0.19	-0.01
Code status							
DNR/partial	114 (15%)	1292 (19%)	137 (26%)	-0.11	0.07	-0.28	0.00
Full Code/Missing	642 (85%)	5373 (81%)	386 (74%)	0.11	-0.07	0.28	0.00
Functional status at discharge							
Non-ambulatory	187 (25%)	1507 (23%)	171 (33%)	0.05	-0.01	-0.18	0.00
Ambulatory	569 (75%)	5158 (77%)	352 (67%)	-0.05	0.01	0.18	0.00
Admission source							
Home/Clinic	626 (83%)	5369 (81%)	431 (82%)	0.06	-0.05	0.01	0.01
Hospital/SNF & Other	130 (17%)	1296 (19%)	92 (18%)	-0.06	0.05	-0.01	-0.01
Anticipated support post discharge							
Self	151 (20%)	1103 (17%)	90 (17%)	0.09	0.08	0.07	0.00
Family/Other/Missing	605 (80%)	5562 (83%)	433 (83%)	-0.09	-0.08	-0.07	0.00
Discharge disposition							
Home/other/missing	554 (75%)	2182 (77%)	345 (66%)	-0.27	-0.05	0.07	0.00
Home health/hospice	188 (25%)	652 (23%)	178 (34%)	0.27	0.05	-0.07	0.00

© 2023 Nguyen HQ et al. *JAMA Health Forum.*

eTable 3. Major Diagnostic Categories for the Non-Heart Failure Cohort

Major diagnostic categories, n(%)	Meals		2019 Control		2021/2022 Control	
Infectious & Parasitic Diseases, Systemic or Unspecified Sites	122	16%	1225	18%	100	19%
Diseases & Disorders of the Circulatory System	133	18%	1019	15%	73	14%
Diseases & Disorders of the Digestive System	118	16%	792	12%	63	12%
Diseases & Disorders of the Respiratory System	89	12%	611	9%	75	14%
Diseases & Disorders of the Musculoskeletal System & Connective Tissue	64	8%	763	11%	39	7%
Diseases & Disorders of the Nervous System	50	7%	563	8%	49	9%
Diseases & Disorders of the Kidney & Urinary Tract	47	6%	367	6%	40	8%
Diseases & Disorders of the Hepatobiliary System & Pancreas	35	5%	265	4%	18	3%
Endocrine, Nutritional & Metabolic Diseases & Disorders	25	3%	190	3%	11	2%
Diseases & Disorders of Blood, Blood Forming Organs, Immunologic Disorders	5	1%	76	1%	11	2%
Myeloproliferative Diseases & Disorders, Poorly Differentiated Neoplasms	9	1%	57	1%	7	1%
Diseases & Disorders of the Skin, Subcutaneous Tissue & Breast	10	1%	122	2%	4	1%
Injuries, Poisonings & Toxic Effects of Drugs	8	1%	47	1%	2	0%
Diseases & Disorders of the Female Reproductive System	6	1%	56	1%	1	0%
Diseases & Disorders of the Male Reproductive System	3	0%	52	1%	4	1%
Other	2	0%	42	1%	4	1%
Mental Diseases & Disorders	2	0%	13	0%	2	0%
Diseases & Disorders of the Ear, Nose, Mouth & Throat	1	0%	31	0%	2	0%
Factors Influencing Health Status & Other Contacts with Health Services	3	0%	23	0%	0	0%
Multiple Significant Trauma	1	0%	7	0%	2	0%
Alcohol/Drug Use & Alcohol/Drug Induced Organic Mental Disorders	1	0%	15	0%	0	0%
Diseases & Disorders of the Eye	1	0%	11	0%	0	0%
Human Immunodeficiency Virus Infections	0	0%	4	0%	0	0%
Pre-MDC	0	0%	10	0%	0	0%
**Total**	**756**		**6665**		**523**	

© 2023 Nguyen HQ et al. *JAMA Health Forum.*

eTable 4. Reasons for Why Eligible Patients Did Not Receive Home-Delivered Meals

	HF	Non-HF	All
Caregiver Will Prepare Meals	51	55	106 (11%)
Client Can Prepare Meals	26	39	65 (7%)
Deceased	6	8	14 (1%)
Dietary Restrictions	12	20	32 (3%)
Long Term Care/Nursing Facility	27	34	61 (6%)
Not Applicable	14	13	27 (3%)
No Contact	171	191	362 (37%)
No Reason Provided/Client Refused	82	87	169 (17%)
Not Enough Space	6	0	6 (1%)
Still in hospital or re-hospitalized	59	69	128 (13%)
Wrong Number	1	1	1 (0%)
Vacation/traveling	0	6	7 (1%)
Total	455	523	978

© 2023 Nguyen HQ et al. *JAMA Health Forum.*

eTable 5. Secondary Descriptive Outcomes at 60-days Post-Discharge

		No Meals Comparators				No Meals Comparators	
	**HF Meals n=742**	**HF 2019 n=2834**	**HF 2021/2022 n=455**		**non-HF Meals n=756**	**non-HF 2019 n=6665**	**non-HF 2021/2022 n=523**
**Post discharge outcomes**							
60-day ED visits	185 (25%)	794 (28%)	136 (30%)		182 (24%)	1710 (26%)	159 (30%)
60-day inpatient/observation stays	230 (31%)	1006 (35%)	189 (42%)		175 (23%)	1633 (25%)	165 (32%)
60-days alive, at home	54.6 (11.73)	51.9 (15.47)	47.9 (18.82)		56.5 (9.23)	54.2 (13.53)	49.1 (18.16)
All-cause outcomes							
Rehospitalization only	181 (24%)	816 (29%)	125 (27%)		153 (20%)	1382 (21%)	119 (23%)
Rehospitalization + death	49 (7%)	190 (7%)	64 (14%)		22 (3%)	251 (4%)	46 (9%)
Death only	21 (3%)	187 (7%)	32 (7%)		14 (2%)	370 (6%)	54 (10%)
None	491 (66%)	1641 (58%)	234 (51%)		567 (75%)	4662 (70%)	304 (58%)
HF-related hospitalizations							
HF-related hospitalizations	87 (12%)	372 (13%)	63 (14%)		-	-	-
HF rehospitalization only	20 (3%)	69 (2%)	30 (7%)		-	-	-
HF rehospitalization + death	50 (7%)	308 (11%)	66 (15%)		-	-	-
None	585 (79%)	2085 (74%)	296 (65%)		-	-	-

© 2023 Nguyen HQ et al. *JAMA Health Forum.*

eTable 6. Secondary Outcomes at 60-days Post-Discharge

**Principal discharge diagnosis: Heart failure**		
**60-day outcomes**	**All-cause death**	**All-cause readmission & death**
HF Meals vs. HF No Meals-2019[a]	OR: 0.89 (0.69, 1.14), P=.36	OR: 0.89 (0.75, 1.05), P=.16
HF Meals vs. HF No Meals-2021/2022[a]	OR: 0.44 (0.30, 0.63), P<.001	OR: 0.61 (0.48, 0.77), P<.001
**Principal discharge diagnosis: All other medical conditions**		
**60-day outcomes**	**All-cause death**	**All-cause readmission & death**
NonHF Meals vs. No Meals-2019[b]	OR: 0.51 (0.36, 0.71), P<.001	OR: 0.74 (0.62, 0.88), P<.001
NonHF Meals vs. No Meals-2021/2022[b]	OR: 0.31 (0.21, 0.46), P<.001	OR: 0.56 (0.44, 0.72), P<.001

[a]Inverse probability of treatment weighting (IPTW) logistic regression models adjusted for age, sex, race/ethnicity, neighborhood deprivation index, prior year acute care utilization, Elixhauser co-morbidity index, ejection fraction, dementia, frailty index, characteristics of the index hospitalization (LACE readmission risk score, length of stay, laboratory acute physiology score, code status, admission source, functional status at discharge, support after discharge, and discharge disposition), and hospital site.
[b]Inverse probability of treatment weighting (IPTW) logistic regression models adjusted for age, sex, race/ethnicity, neighborhood deprivation index, prior year acute care and ambulatory utilization, ejection fraction, dementia, frailty index, exercise in prior year, characteristics of the index hospitalization (LACE readmission risk score, length of stay, laboratory acute physiology score, code status, admission source, functional status at discharge, support after discharge, and discharge disposition), and hospital site.

© 2023 Nguyen HQ et al. *JAMA Health Forum.*

eFigure 1. Days to Rehospitalization and Death

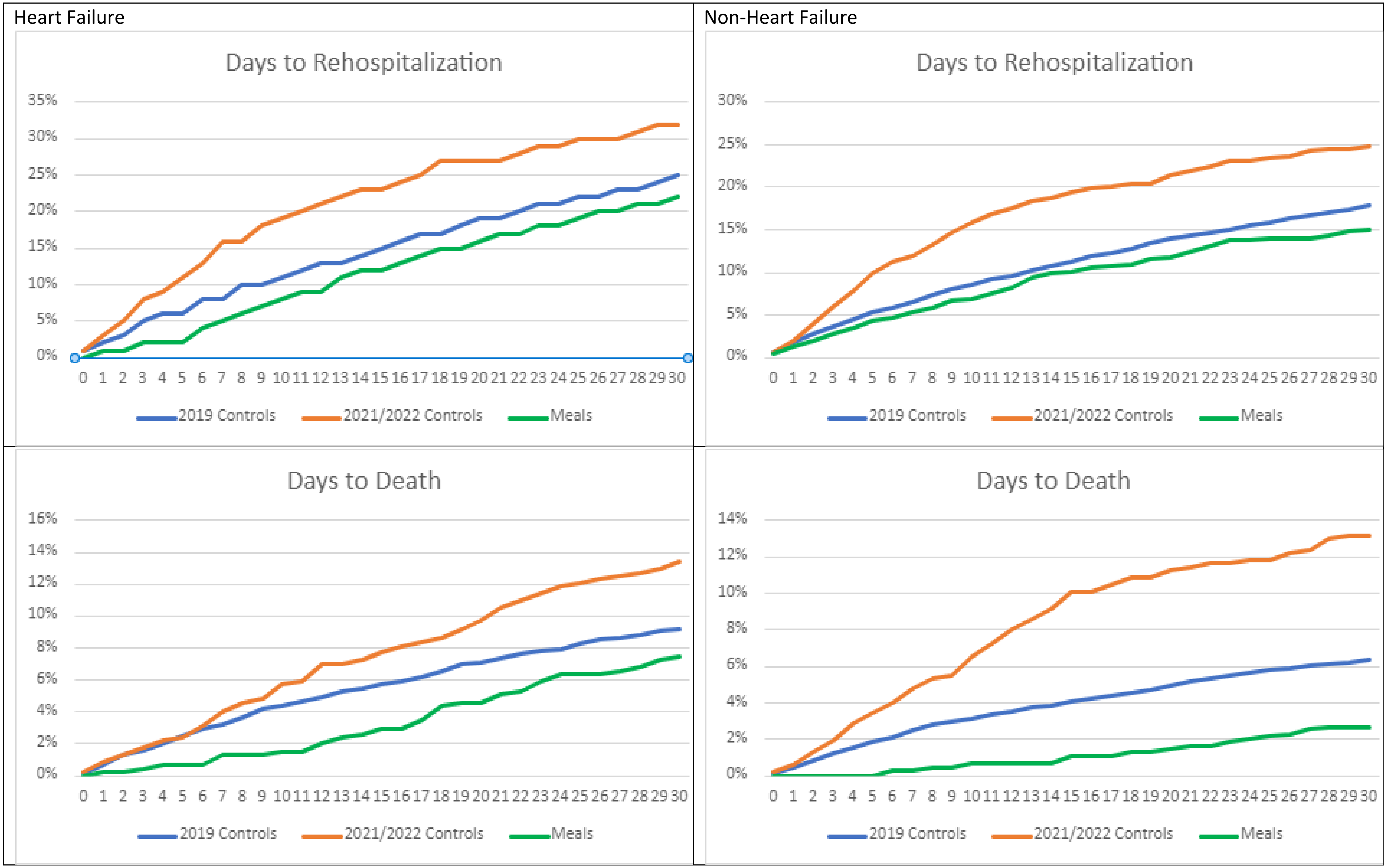

© 2023 Nguyen HQ et al. *JAMA Health Forum.*
