# Supplementary material for: Association of a Medicare Advantage Posthospitalization Home Meal Delivery Benefit With Rehospitalization and Death
Source: JAMA Health Forum. 2023 Jun 25;4(6):e231678. doi: 10.1001/jamahealthforum.2023.1678 (PMC10291360; doi:10.1001/jamahealthforum.2023.1678)
Supplement: Supplement 2. — Data Sharing Statement [file jamahealthforum-e231678-s002.pdf]

Data Sharing Statement

Nguyen. Association of a Medicare Advantage Posthospitalization Home Meal Delivery Benefit With Rehospitalization and Death. *JAMA Health Forum*. Published June 25, 2023. doi:10.1001/jamahealthforum.2023.1678

Data
Data available: No

Additional Information
Explanation for why data not available: Data can be made available with request to Dr. Nguyen
